# Time representation in reinforcement learning models of the basal ganglia

**DOI:** 10.3389/fncom.2013.00194

**Published:** 2014-01-09

**Authors:** Samuel J. Gershman, Ahmed A. Moustafa, Elliot A. Ludvig

**Affiliations:** ^1^Department of Brain and Cognitive Sciences, Massachusetts Institute of TechnologyCambridge, MA, USA; ^2^School of Social Sciences and Psychology, Marcs Institute for Brain and Behaviour, University of Western SydneySydney, NSW, Australia; ^3^Princeton Neuroscience Institute and Department of Mechanical and Aerospace Engineering, Princeton UniversityPrinceton, NJ, USA; ^4^Department of Psychology, University of WarwickCoventry, UK

**Keywords:** reinforcement learning, basal ganglia, dopamine, interval timing, Parkinson's disease

## Abstract

Reinforcement learning (RL) models have been influential in understanding many aspects of basal ganglia function, from reward prediction to action selection. Time plays an important role in these models, but there is still no theoretical consensus about what kind of time representation is used by the basal ganglia. We review several theoretical accounts and their supporting evidence. We then discuss the relationship between RL models and the timing mechanisms that have been attributed to the basal ganglia. We hypothesize that a single computational system may underlie both RL and interval timing—the perception of duration in the range of seconds to hours. This hypothesis, which extends earlier models by incorporating a time-sensitive action selection mechanism, may have important implications for understanding disorders like Parkinson's disease in which both decision making and timing are impaired.

## Introduction

Computational models of reinforcement learning (RL) have had a profound influence on the contemporary understanding of the basal ganglia (Joel et al., 2002; Cohen and Frank, 2009). The central claim of these models is that the basal ganglia are organized to support prediction, learning and optimization of long-term reward. While this claim is now widely accepted, RL models have had little to say about the extensive research implicating the basal ganglia in interval timing—the perception of duration in the range of seconds to hours (Buhusi and Meck, 2005; Jones and Jahanshahi, 2009; Merchant et al., 2013). However, this is not to say that time is ignored by these models—on the contrary, time representation has been a pivotal issue in RL theory, particularly with regard to the role of dopamine (Suri and Schultz, 1999; Daw et al., 2006; Ludvig et al., 2008; Nakahara and Kaveri, 2010; Rivest et al., 2010).

In this review, we attempt a provisional synthesis of research on RL and interval timing in the basal ganglia. We begin by briefly reviewing RL models of the basal ganglia, with a focus on how they represent time. We then summarize the key data linking the basal ganglia with interval timing, drawing connections between computational approaches to timing and their relationship to RL models. Our central thesis is that by incorporating a time-sensitive action selection mechanism into RL models, a single computational system can support both RL and interval timing. This unified view leads to a coherent interpretation of decision making and timing deficits in Parkinson's disease.

## Reinforcement learning models of the basal ganglia

RL models characterize animals as agents that seek to maximize future reward (for reviews, see Maia, 2009; Niv, 2009; Ludvig et al., 2011). To do so, animals are assumed to generate a prediction of future reward and select actions according to a policy that maximizes that reward. More formally, suppose that at time *t* an agent occupies a state *s*_*t*_ (e.g., the agent's location or the surrounding stimuli) and receives a reward *r*_*t*_. The agent's goal is to predict the *expected discounted future return*, or *value*, of visiting a sequence of states starting in state *s*_*t*_ (Sutton and Barto, 1998):

(1) $$V(S_{t})=E\left[\sum_{k=0}\gamma^{k}r_{t+k}\right],$$

where γ is a parameter that discounts distal rewards relative to proximal rewards, and *E* denotes an average over possibly stochastic sequences of states and rewards.

Typically, a state *s*_*t*_ is described by a set of *D* features, {*x*_*t*_ (1), …, *x*_*t*_ (*D*)}, encoding sensory and cognitive aspects of an animal's current experience. Given this state representation, the value can be approximated by a weighted combination of the features:

$$\hat{V}\left(s_{t}\right)=\sum_{d}w_{t}\left(d\right)x_{t}\left(d\right)$$

where $\hat{V}$ is an estimate of the true value *V*. According to RL models of the basal ganglia, these features are represented by cortical inputs to the striatum, with the striatum itself encoding the estimated value (Maia, 2009; Niv, 2009; Ludvig et al., 2011). The strengths of these corticostriatal synapses are represented by a set of weights {*w*_*t*_ (1), …, *w*_*t*_ (*D*)}.

These weights can be learned through a simple algorithm known as *temporal-difference (TD) learning*, which adjusts the weights on each time step based on the difference between received and predicted reward:

$$w_{t+1}\left(d\right)=w_{t}\left(d\right)+\alpha \delta_{t}e_{t}\left(d\right),$$

where α is a learning rate and δ_*t*_ is a prediction error defined as:

$$\delta_{t}=r_{t}+\gamma \hat{V}\left(s_{t+1}\right)-\hat{V}\left(s_{t}\right).$$

The *eligibility trace e*_*t*_(*d*) is updated according to:

$$e_{t+1}\left(d\right)=\gamma \lambda e_{t}\left(d\right)+x_{t}(d),$$

where λ is a decay parameter that determines the plasticity window of recent stimuli. The TD algorithm is a computationally efficient method that is known to converge to the true value function [see Equation (1) above] with enough experience and adequate features (Sutton and Barto, 1998).

The importance of this algorithm to neuroscience lies in the fact that the firing of midbrain dopamine neurons conforms remarkably well to the theoretical prediction error (Houk et al., 1995; Montague et al., 1996; Schultz et al., 1997; though see Redgrave et al., 2008 for a critique). For example, dopamine neurons increase their firing upon the delivery of an unexpected reward and pause when an expected reward is omitted (Schultz et al., 1997). The role of prediction errors in learning is supported by the observation that plasticity at corticostriatal synapses is gated by dopamine (Reynolds and Wickens, 2002; Steinberg et al., 2013), as well as a large body of behavioral evidence (Rescorla and Wagner, 1972; Sutton and Barto, 1990; Ludvig et al., 2012).

A fundamental question facing RL models is the choice of feature representation. Early applications of TD learning to the dopamine system assumed what is known as the *complete serial compound* (CSC; Moore et al., 1989; Sutton and Barto, 1990; Montague et al., 1996; Schultz et al., 1997), which represents every time step following stimulus onset as a separate feature. Thus, the first feature has a value of 1 for the first time step and 0 for all other time steps, the second feature has a value of 1 for the second time step and 0 for all other time steps, and so on. This CSC representation assumes a perfect clock, whereby the brain always knows exactly how many time steps have elapsed since stimulus onset.

The CSC is effective at capturing several salient aspects of the dopamine response to cued reward. A number of authors (e.g., Daw et al., 2006; Ludvig et al., 2008), however, have pointed out aspects of the dopamine response that appear inconsistent with the CSC. For example, the CSC predicts a large, punctate negative prediction error when an expected reward is omitted; the actual decrease in dopamine response is relatively small and temporally extended (Schultz et al., 1997; Bayer et al., 2007). Another problem with the CSC is that it predicts a large negative prediction error at the usual reward delivery time when a reward is delivered early. Contrary to this prediction, Hollerman and Schultz (1998) found that early reward evoked a large response immediately after the unexpected reward, but showed little change from baseline at the usual reward delivery time.

It is possible that these mismatches between theory and data reflect problems with a number of different theoretical assumptions. Indeed, several theoretical assumptions have been questioned by recent research (see Niv, 2009). We focus here on alternative time representations as one potential response to the findings mentioned above.

**Figure 1:**
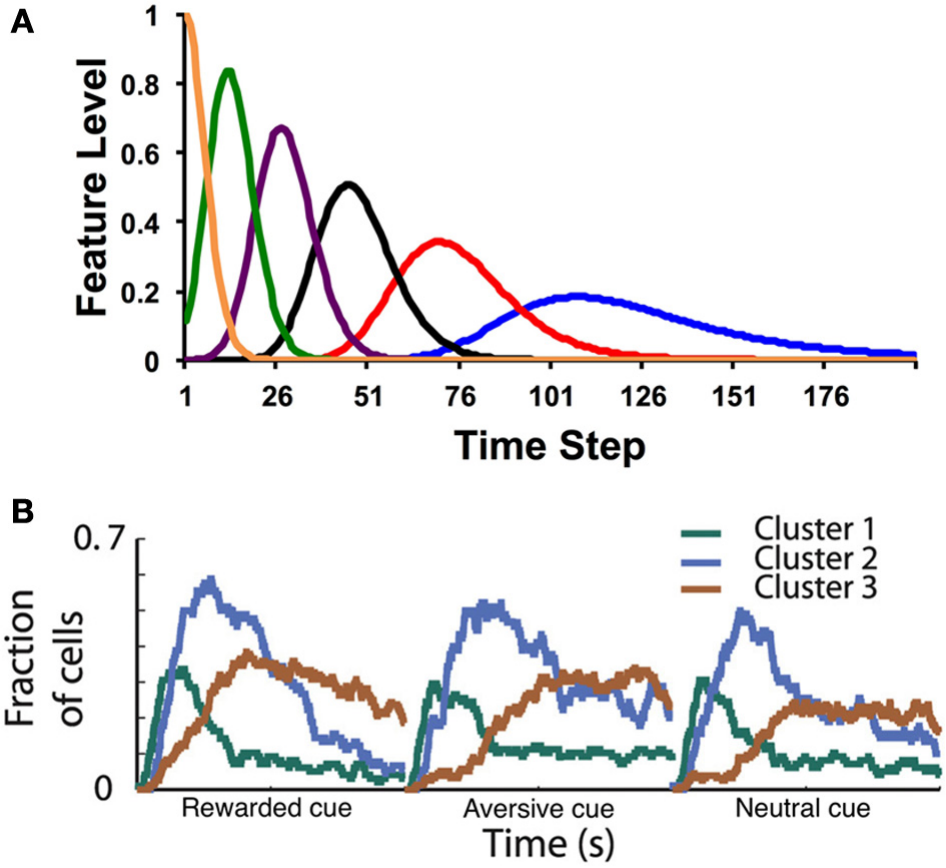
**(A)** Example microstimuli. Each microstimulus acts as a dynamic feature, going up and then coming back down with a characteristic time course after the stimulus. **(B)** Fraction of medium spiny neurons recorded in the putamen with a significant response (both increases and decreases in firing rate) to cue events. In this task, a visual cue (fractal image) is presented at time 0, followed by an outcome (food, air puff, or neutral sound) 2 s later. Clusters correspond to groups of neurons with similar response profiles (see Adler et al., 2012 for details). Time courses are shown separately for cues that predict rewarding (food), aversive (air puff), or neutral (sound) outcomes. Note the similarity in the population response to the postulated time course of the theoretical microstimuli. Figure adapted from Adler et al. (2012) with permission from the publisher.

We will discuss two of these alternatives (see also Suri and Schultz, 1999; Nakahara and Kaveri, 2010; Rivest et al., 2010): (1) the microstimulus representation and (2) states with variable durations (a *semi-Markov* formalism) and only partial observability. For the former, Ludvig et al. (2008) proposed that when a stimulus is presented, it leaves a slowly decaying memory trace, which is encoded by a series of temporal receptive fields. Each feature (or “microstimulus”) *x*_*t*_(*d*) represents the proximity between the trace and the center of the receptive field, producing a spectrum of features that vary with time, as illustrated in Figure 1A. Specifically, Ludvig et al. endowed each stimulus with microstimuli of the following form:

$$x_{t}(d)=\frac{y_{t}}{\sqrt{\sigma^{2}}}\exp\left(-\frac{{\left(y_{t}-\frac{d}{D}\right)}^{2}}{2\sigma^{2}}\right)$$

where *D* is the number of microstimuli, σ^2^ controls the width of each receptive field, and *y*_*t*_ is the stimulus trace strength, which was set to 1 at stimulus onset and decreased exponentially with a decay rate of 0.985 per time step. Both cues and rewards elicit their own set of microstimuli. This feature representation is plugged into the TD learning equations described above.

The microstimulus representation is a temporally smeared version of the CSC: whereas in the CSC each feature encodes a single time point, in the microstimulus representation each feature encodes a temporal range (see also Grossberg and Schmajuk, 1989; Machado, 1997). With the CSC, as time elapses after a stimulus, there is one, unique feature active at each time point. Learned weights for that time point therefore all accrue to that one feature. In contrast, at any time point, a subset of the microstimuli is differentially activated. These serve as the features that can be used to generate a prediction of upcoming reward (values). Note how the temporal precision of the microstimuli decreases with time following stimulus onset, so that later microstimuli are more dispersed than earlier microstimuli.

Recent data from Adler et al. (2012) have provided direct evidence for microstimuli in the basal ganglia (Figure 1B). Recording from the putamen while a monkey was engaged in a classical conditioning task, Adler et al. found clusters of medium spiny neurons with distinct post-stimulus time courses (for both cues and outcomes). As postulated by Ludvig et al. (2008), the peak response time varied across clusters, with long latency peaks (i.e., late microstimuli) associated with greater dispersion. Recording from the caudate nucleus, Jin et al. (2009) also found clusters of neurons that encode time-stamps of different events. These neurons carry sufficient information to decode time from the population response. Early time points are decodable with higher fidelity compared to late time points, as would be expected if the dispersion of temporal receptive fields increases with latency.

A different solution to the limitations of the CSC was suggested by Daw et al. (2006). They proposed that dopaminergic prediction errors reflect a state space that is *partially observable* and *semi-Markov*. The partial-observability assumption means that the underlying state is inferred from sensory data (cues and rewards), rather than using the features as a proxy for the state. Thus, prediction errors are computed with respect to a *belief state*, a set of features encoding the probabilistic inference about the hidden state. The semi-Markov assumption means that each state is occupied for a random amount of time before transitioning. In the simulations of Daw et al. (2006), only two states were postulated: an interstimulus interval (ISI) state and an intertrial interval (ITI) state. Rewards are delivered upon transition from the ISI to the ITI state, and cues occur upon transition from the ITI to the ISI state. The learning rule in this model is more complex than the standard TD learning rule [which is used by the Ludvig et al. (2008) model]; however, the core idea of learning from prediction errors is preserved in this model.

It is instructive to compare how these two models account for the data on early reward presented by Hollerman and Schultz (1998). In the Ludvig et al. (2008) model, the weights for all the microstimuli are updated after every time step: the late microstimuli associated with the cue (i.e., those centered around the time of reward delivery) accrue positive weights, even after the time of reward delivery (a consequence of the temporal smearing). These post-reward positive predictions generate a negative prediction error, causing the early microstimuli associated with the reward to accrue negative weights. When reward is presented early, the net prediction is close to zero, because the positive weights on the late cue microstimuli compete with the negative weights on the early reward microstimuli. This interaction produces a negligible negative prediction error, consistent with the data of Hollerman and Schultz (1998). The account of Daw et al. (2006) is conceptually different: when reward is presented early, the model infers that a transition to the ITI state has occurred early, and consequently no reward is expected.

Thus far, we have discussed time representations in the service of RL and their implications for the timing of the dopamine response during conditioning. What do RL models have to say about interval timing *per se*? We will argue below that these are not really separate problems: interval timing tasks can be viewed fundamentally as RL tasks. Concomitantly, the role of dopamine and the basal ganglia in interval timing can be understood in terms of their computational contributions to RL. To elaborate this argument, we need to first review some of the relevant theory and data linking interval timing with the basal ganglia.

## Time representation in the basal ganglia: data and theory

**Figure 2:**
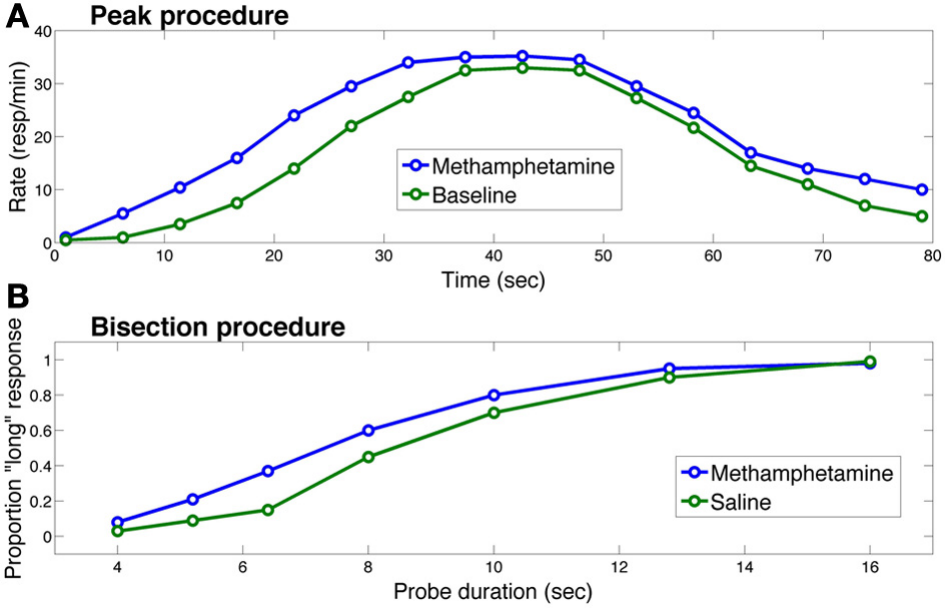
**Effects of methamphetamine on timing in (A) the peak procedure and (B) the bisection procedure in rats**. Each curve in **(A)** represents response rate as a function of time, where time 0 corresponds to the trial onset. The methamphetamine curve corresponds to sessions in which rats were injected with methamphetamine; the baseline curve corresponds to sessions in which rats did not receive an injection. Each curve in **(B)** represents the proportion of trials on which the rat chose the “long” option as a function of probe cue duration. The saline curve corresponds to sessions in which the rat received a saline injection. In both procedures, methamphetamine leads to overestimation of the elapsing interval, producing early responding in the peak procedure and more “long” responses in the bisection procedure. Figure replotted from Maricq et al. (1981).

The role of the basal ganglia and dopamine in interval timing has been studied most extensively in the context of two procedures: the peak procedure (Catania, 1970; Roberts, 1981) and the bisection procedure (Church and Deluty, 1977). The peak procedure consists of two trial types: on fixed-interval trials, the subject is rewarded if a response is made after a fixed duration following cue presentation. On probe trials, the cue duration is extended, and no reward is delivered for responding. Figure 2A shows a typical response curve on probe trials: on average, the response rate peaks around the time of food presentation (20 or 40 s in the figure) is ordinarily available and then decreases. The peak time (a measure of the animal's interval estimate) is the time at which the response rate is maximal.

The other two curves in Figure 2A illustrate the standard finding that drugs (or genetic manipulations) that increase dopamine transmission, such as methamphetamine, shift the response curve leftward (Maricq et al., 1981; Matell et al., 2004, 2006; Cheng et al., 2007; Balci et al., 2010), whereas drugs that decrease dopamine transmission shift the response curve rightward (Drew et al., 2003; Macdonald and Meck, 2005).

In the bisection procedure, subjects are trained to respond differentially to short and long duration cues. Unreinforced probe trials with cue durations between these two extremes are occasionally presented. On these trials, typically, a psychometric curve is produced with greater selection of the long option (i.e., the option reinforced following long duration cues) with longer probes and greater selection of the short option with shorter probes and a gradual shift between the two (see Figure 2B). The indifference point or point of subjective equality is typically close to the geometric mean of the two anchor durations (Church and Deluty, 1977). Similar to the peak procedure, in the bisection procedure, Figure 2B shows how dopamine agonists usually produce a leftward shift in the psychometric curve—i.e., more “short” responses, whereas dopamine antagonists produce the opposite pattern (Maricq et al., 1981; Maricq and Church, 1983; Meck, 1986; Cheng et al., 2007). Under some circumstances, however, dopamine agonists induce temporal dysregulation with an overall flattening of the response curve and no shift in preference or peak times (e.g., Odum et al., 2002; McClure et al., 2005; Balci et al., 2008).

The most influential interpretation of these findings draws upon the class of pacemaker-accumulator models (Gibbon et al., 1997), according to which a pacemaker (an “internal clock”) emits pulses that are accumulated by a counter to form a representation of subjective time intervals. The neurobiological implementation of this scheme might rely on populations of oscillating neurons (Miall, 1989; Matell and Meck, 2004), integration of ramping neural activity (Leon and Shadlen, 2003; Simen et al., 2011), or intrinsic dynamics of a recurrent network (Buonomano and Laje, 2010). Independent of the neural implementation, the idea is that drugs that increase dopamine speed up the internal clock, while drugs that decrease dopamine slow the internal clock down.

This interpretation is generally consistent with the findings from studies of patients with Parkinson's disease (PD), who have chronically low striatal dopamine levels. When off medication, these patients tend to underestimate the length of temporal intervals in verbal estimation tasks; dopaminergic medication alleviates this underestimation (Pastor et al., 1992; Lange et al., 1995). It should be noted, however, that some studies have found normal time perception in PD (Malapani et al., 1998; Spencer and Ivry, 2005; Wearden et al., 2008), possibly due to variations in disease severity (Artieda et al., 1992).

Pacemaker-accumulator models have been criticized on a number of grounds, such as lack of parsimony, implausible neurophysiological assumptions, and incorrect behavioral predictions (Staddon and Higa, 1999, 2006; Matell and Meck, 2004; Simen et al., 2013). Moreover, while the pharmacological data are generally consistent with the idea that dopamine modulates the speed of the internal clock, these data may also be consistent with other interpretations. One important alternative is the class of “distributed elements” models, which postulate a representation of time that is distributed over a set of elements; these elements come in various flavors, such as “behavioral states” (Machado, 1997), a cascade of leaky integrators (Staddon and Higa, 1999, 2006; Shankar and Howard, 2012), or spectral traces (Grossberg and Schmajuk, 1989). The effects of dopaminergic drugs might be explicable in terms of systematic changes in the pattern of activity across the distributed elements (see, for example, Grossberg and Schmajuk, 1989).

In fact, the microstimulus model of Ludvig et al. (2008) can be viewed as a distributed elements model embedded within the machinery of RL. This connection suggests a more ambitious theoretical synthesis: can we understand the behavioral and neurophysiological characteristics of interval timing in terms of RL?

## Toward a unified model of reinforcement learning and timing

**Figure 3:**
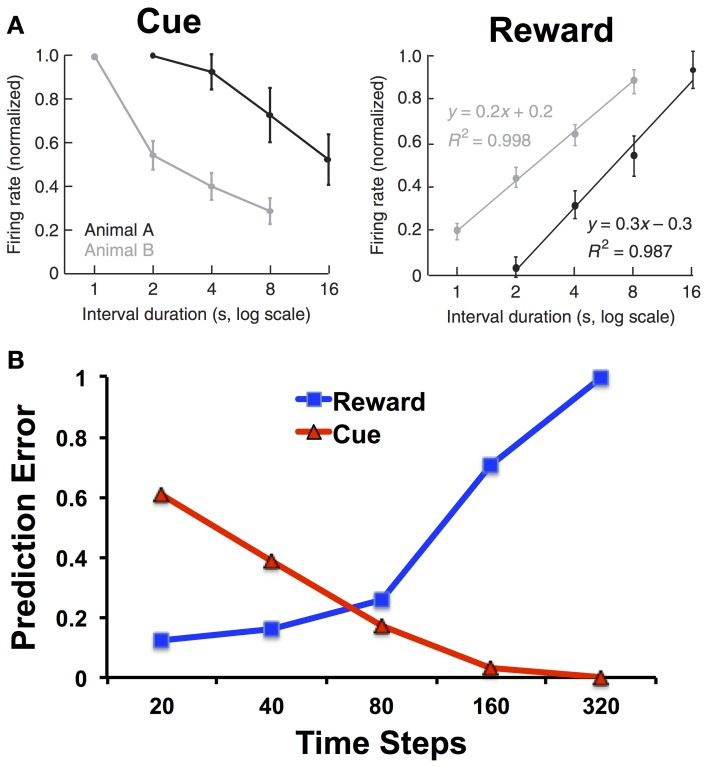
**(A)** Firing rates of dopamine neurons in monkeys to cues and rewards as a function of cue-reward interval duration. Adapted from Fiorillo et al. (2008) with permission from the publisher. **(B)** Prediction errors to the reward and cue as a function of the interval duration for the microstimulus model. The simulations of the microstimulus model were run for 100 trials and used the same parameters specified in Ludvig et al. (2008): λ = 0.95, α = 0.01, γ = 0.98, *D* = 50, σ = 0.08. We treated 20 time steps as a unit of 1 s, and each trial was separated by an interval of 500 time steps. Note the logarithmic scale on the x-axis.

One suggestive piece of evidence for how RL models and interval timing can be integrated comes from the study of Fiorillo et al. (2008); (see also Kobayashi and Schultz, 2008). They trained monkeys on a variation of the peak procedure with classical contingencies (i.e., water was delivered independent of responding) while recording from dopamine neurons in the substantia nigra and ventral tegmental area with five different intervals spanning from 1 to 16 s. As shown in Figure 3A, they found that the dopamine response to the reward increased with the interval, and the dopamine response to the cue decreased with the interval.

Whereas the response to the cue can be explained in terms of temporal discounting, the response to the reward should not (according to the CSC representation) depend on the cue-reward interval. The perfect timing inherent in the CSC representation means that the reward can be equally well predicted at all time points. Thus, there should be no reward-prediction error, and no phasic dopamine response, at the time of reward regardless of the cue-reward interval. Alternatively, the dopamine response to reward can be understood as reflecting increasing uncertainty in the temporal prediction. Figure 3B shows how, using the microstimulus TD model as defined as in Ludvig et al. (2008), there is indeed an increase in the simulated reward prediction error as a function of interval. In the model, with longer intervals, the reward predictions are less temporally precise, and greater prediction errors persist upon reward receipt.

Interval timing procedures, such as the peak procedure, add an additional nuance to this problem by introducing instrumental contingencies. Animals must now not only predict the timing of reward, but also learn when to respond. To analyze this problem in terms of RL, we need to augment the framework introduced earlier to have actions. There are various ways to accomplish this (see Sutton and Barto, 1998). The Actor-Critic architecture (Houk et al., 1995; Joel et al., 2002) is one of the earliest and most influential approaches; it postulates a separate “actor” mechanism that probabilistically chooses an action *a*_*t*_ given the current state *s*_*t*_. The action probabilities *p*(*s*_*t*_|*a*_*t*_) ∝ exp{*>f*(*s*_*t*_|*a*_*t*_)} are updated according to:

$$f(s_{t}|a_{t})\leftarrow f(s_{t}|a_{t})+\eta \delta_{t}\left[1-p\left(s_{t}|a_{t}\right)\right],$$

where η is a learning rate parameter and δ_*t*_ is the is the prediction error defined earlier. The value estimation system plays the role of a “critic” that teaches the actor how to modify its action selection probabilities so as to reduce prediction errors.

When combined with the microstimulus representation, the actor-critic architecture naturally gives rise to timing behavior: in the peak procedure, on average, responding will tend to increase toward the expected reward time and decrease thereafter (see Figure 2). Importantly, the late microstimuli are less temporally precise than the early microstimuli, in the sense that their responses are more dispersed over time. As a consequence, credit for late rewards is assigned to a larger number of microstimuli. Under the assumption that response rate is proportion to predicted value, this dispersion of credit causes the timing of actions to be more spread out around the time of reward as the length of the interval increases, one of the central empirical regularities in timing behavior (Gibbon, 1977; see also Ludvig et al., 2012 for an exploration of this property in classical conditioning). As described above, an analog of this property has also been observed in the firing of midbrain dopamine neurons: response to reward increases linearly with the logarithm of the stimulus-reward interval, consistent with the idea that prediction errors are being computed with respect to a value signal whose temporal precision decreases over time (Fiorillo et al., 2008). To the best of our knowledge, pacemaker-accumulator models cannot account for the results presented in Figure 3, because they do not have reward-prediction errors in their machinery. Instead, they collect a distribution of past cue-reward intervals and draw from that distribution to create an estimate of the time to reward (e.g., Gibbon et al., 1984).

**Figure 4:**
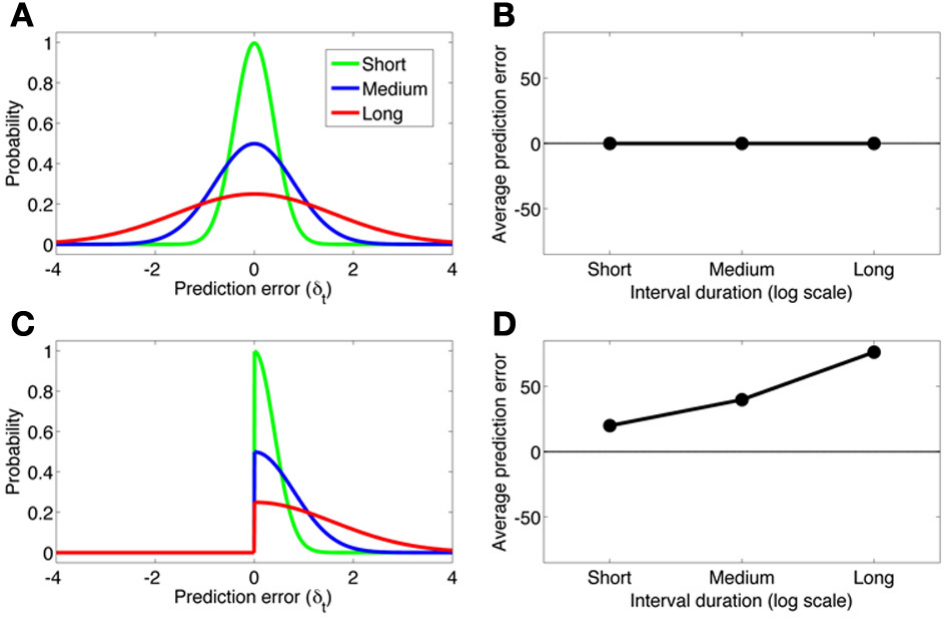
**(A)** The distribution of prediction errors in the semi-Markov TD model (Daw et al., 2006) for three logarithmically spaced cue-reward interval durations (short, medium, long). The timing signal is corrupted by zero-mean Gaussian noise with standard deviation proportional to the interval duration. **(B)** The average prediction error under each distribution. A symmetric prediction error distribution implies that the average prediction error is invariant to the interval duration. **(C,D)** Same format as panels A and B, but where the prediction error distribution is rectified slightly below 0. This asymmetric distribution produces average prediction errors that scale approximately linearly with the log interval duration.

The partially observable semi-Markov model of Daw et al. (2006) can account for the findings of Fiorillo et al. (2008), but this account deviates from the normative RL framework. Daw et al. use an external timing signal with “scalar noise” (cf. Gibbon et al., 1984), implemented by adding Gaussian noise to the timing signal with standard deviation proportional to the interval. Scalar noise induces larger-magnitude prediction errors with increasing delays. However, these prediction errors are symmetric around 0 and hence cancel out on average. To account for the effects of cue-reward interval on the dopamine response, Daw et al. assume that negative prediction errors are rectified (see Bayer and Glimcher, 2005), resulting in a positive skew of the prediction error distribution. Figure 4 shows how this asymmetric rectification results in average prediction errors that are increasingly positive for longer intervals. Note that rectification is not an intrinsic part of the RL framework, and in fact compromises the convergence of TD to the true value function. One potential solution to this problem is to posit a separate physiological channel for the signaling of negative prediction errors, possibly via serotonergic activity (Daw et al., 2002).

The microstimulus actor-critic model can also explain the effects of dopamine manipulations and Parkinson's disease. The key additional assumption is that early microstimuli (but not later ones) are primarily represented by the striatum. Timing in the milliseconds to seconds range depends on D2 receptors in the dorsal striatum (Rammsayer, 1993; Coull et al., 2011), suggesting that this region represents early microstimuli (whereas late microstimuli may be represented by other neural substrates, such as the hippocampus; see Ludvig et al., 2009). Because the post-synaptic effect of dopamine at D2 receptors is inhibitory, D2 receptor antagonists increase the firing of striatal neurons expressing D2 receptors, which mainly occur in the indirect or “NoGo” pathway and exert a suppressive influence on striatal output (Gerfen, 1992). Thus, the ultimate effect of D2 receptor antagonists is to reduce striatal output, thereby attenuating the influence of early microstimuli on behavior. As a result, predictions of the upcoming reward will be biased later, and responses will occur later than usual (e.g., in the peak procedure). This fits with the observation that the rightward shift (overestimation) of estimated time following dopamine antagonist administration is proportional to the drug's binding affinity for D2 receptors (Meck, 1986). In contrast, dopamine agonists lead to a selective enhancement of the early microstimuli, producing earlier than usual responding (see Figure 2A).

A similar line of reasoning can explain some of the timing deficits in Parkinson's disease. The nigrostriatal pathway (the main source of dopamine to the dorsal striatum) is compromised in Parkinson's disease, resulting in reduced striatal dopamine levels. Because D2 receptors have a higher affinity for dopamine, Parkinson's disease leads to the predominance of D2-mediated activity and hence reduced striatal output (Wiecki and Frank, 2010). Our model thus predicts a rightward shift of estimated time, as is often observed experimentally (see above).

The linking of early microstimuli with the striatum in the model also leads to the prediction that low striatal dopamine levels will result in poorer learning of fast responses (which depend on the early microstimuli). In addition, responding will in general be slowed because the learned weights to the early microstimuli will be weak relative to those of late microstimuli. As a result, our model clearly predicts poorer learning of fast responses in Parkinson's disease. A study of temporal decision making in Parkinson's patients fits with this prediction (Moustafa et al., 2008). Patients were trained to respond at different latencies to a set of cues, with slow responses yielding more reward in an “increasing expected value” (IEV) condition and fast responses yielding more reward in a “decreasing expected value” (DEV) condition. It was found that the performance of medicated patients was better in the DEV condition, while performance of non-medicated patients was better in the IEV condition. If non-medicated patients have a paucity of short-timescale microstimuli (due to low striatal dopamine levels), then the model correctly anticipates that these patients will be impaired at learning about early events relative to later events.

Recently, Foerde et al. (2013) found that Parkinson's patients are impaired in learning from immediate, but not delayed, feedback in a probabilistic decision making task. This finding is also consistent with the idea that these patients lack a representational substrate for early post-stimulus events. Interestingly, they also found that patients with lesions of the medial temporal lobe show the opposite pattern, possibly indicating that this region (and in particular the hippocampus) is important for representing late microstimuli, as was suggested earlier by Ludvig et al. (2009). An alternative possibility is that the striatum and hippocampus both represent early and late microstimuli, but the stimulus trace decays more quickly in the striatum than in the hippocampus (Bornstein and Daw, 2012), which would produce a more graded segregation of temporal sensitivity between the two regions.

## Conclusion

Timing and RL have for the most part been studied separately, giving rise to largely non-overlapping computational models. We have argued here, however, that these models do in fact share some important commonalities and reconciling them may provide a unified explanation of many behavioral and neural phenomena. While in this brief review we have only sketched such a synthesis, our goal is to plant the seeds for future theoretical unification.

One open question concerns how to reconcile the disparate theoretical ideas about time representation that were described in this paper. Our synthesis proposed a central role for a distributed elements representation of time such as the microstimuli of Ludvig et al. (2008). Could a representation deriving from the semi-Markov or pacemaker-accumulator models be used instead? This may be possible, but there are several reasons to prefer the microstimulus representation. First, microstimuli lend themselves naturally to the linear function approximation architecture that has been widely used in RL models of the basal ganglia. In contrast, the semi-Markov model requires additional computational machinery, and it is not obvious how to incorporate the pacemaker-accumulator model into RL theory. Second, the semi-Markov model accounts for the relationship between temporal precision and interval length at the expense of deviating from the normative RL framework. Third, as we noted earlier, pacemaker-accumulator models have a number of other weaknesses (see Staddon and Higa, 1999, 2006; Matell and Meck, 2004; Simen et al., 2013), such as lack of parsimony, implausible neurophysiological assumptions, and incorrect behavioral predictions. Nonetheless, it will be interesting to explore what aspects of these models can be successfully incorporated into the next generation of RL models.

### Conflict of interest statement

The authors declare that the research was conducted in the absence of any commercial or financial relationships that could be construed as a potential conflict of interest.
